# A Loss-of-Function *HCN4* Mutation Associated With Familial Benign Myoclonic Epilepsy in Infancy Causes Increased Neuronal Excitability

**DOI:** 10.3389/fnmol.2018.00269

**Published:** 2018-08-06

**Authors:** Giulia Campostrini, Jacopo C. DiFrancesco, Barbara Castellotti, Raffaella Milanesi, Tomaso Gnecchi-Ruscone, Mattia Bonzanni, Annalisa Bucchi, Mirko Baruscotti, Carlo Ferrarese, Silvana Franceschetti, Laura Canafoglia, Francesca Ragona, Elena Freri, Angelo Labate, Antonio Gambardella, Cinzia Costa, Cinzia Gellera, Tiziana Granata, Andrea Barbuti, Dario DiFrancesco

**Affiliations:** ^1^Molecular Physiology and Neurobiology, The PaceLab, Department of Biosciences, Università degli Studi di Milano, Milan, Italy; ^2^Clinical Neurophysiology and Epilepsy Center, Fondazione IRCCS Istituto Neurologico Carlo Besta, Milan, Italy; ^3^Laboratory of Neurobiology, Department of Neurology, Milan Center for Neuroscience, San Gerardo Hospital, University of Milano-Bicocca, Monza, Italy; ^4^Unit of Genetics of Neurodegenerative and Metabolic Diseases, Fondazione IRCCS Istituto Neurologico Carlo Besta, Milan, Italy; ^5^Department of Cardiology, San Leopoldo Mandic Hospital, Merate, Italy; ^6^Department of Pediatric Neuroscience, Fondazione IRCCS Istituto Neurologico Carlo Besta, Milan, Italy; ^7^Institute of Neurology, Università degli Studi Magna Græcia di Catanzaro, Catanzaro, Italy; ^8^Neurology Unit, Ospedale S. Maria della Misericordia, Department of Medicine, University of Perugia, Perugia, Italy

**Keywords:** HCN4, epilepsy, ion channels, myoclonic epilepsy of infancy, neuronal excitability

## Abstract

HCN channels are highly expressed and functionally relevant in neurons and increasing evidence demonstrates their involvement in the etiology of human epilepsies. Among HCN isoforms, HCN4 is important in cardiac tissue, where it underlies pacemaker activity. Despite being expressed also in deep structures of the brain, mutations of this channel functionally shown to be associated with epilepsy have not been reported yet. Using Next Generation Sequencing for the screening of patients with idiopathic epilepsy, we identified the p.Arg550Cys (c.1648C>T) heterozygous mutation on *HCN4* in two brothers affected by benign myoclonic epilepsy of infancy. Functional characterization in heterologous expression system and in neurons showed that the mutation determines a loss of function of HCN4 contribution to activity and an increase of neuronal discharge, potentially predisposing to epilepsy. Expressed in cardiomyocytes, mutant channels activate at slightly more negative voltages than wild-type (WT), in accordance with borderline bradycardia. While HCN4 variants have been frequently associated with cardiac arrhythmias, these data represent the first experimental evidence that functional alteration of HCN4 can also be involved in human epilepsy through a loss-of-function effect and associated increased neuronal excitability. Since HCN4 appears to be highly expressed in deep brain structures only early during development, our data provide a potential explanation for a link between dysfunctional HCN4 and infantile epilepsy. These findings suggest that it may be useful to include *HCN4* screening to extend the knowledge of the genetic causes of infantile epilepsies, potentially paving the way for the identification of innovative therapeutic strategies.

## Introduction

The genetic causes of epilepsy are unknown in the vast majority of patients, although the disease often affects more than one member of the family, suggesting a genetically based disease. Studies investigating the etiology of generalized epilepsies often reveal complex and multifactorial inheritance features (Helbig et al., 2008). A recent genome-wide study, for example, has reported broad locus heterogeneity in generalized epilepsies (Epi4K Consortium and Epilepsy Phenome/Genome Project, 2017).

Nevertheless, a small number of causative genetic alterations have indeed been identified in patients. The most frequent mutations proposed to be involved in generalized epilepsy have been observed in the *SLC2A1* gene, responsible for the Glucose transporter type 1-deficiency syndrome and account up to 0.5%–1% of patients (Arsov et al., 2012; Striano et al., 2012). Other genetic causes involve the dysfunction of ion channels, mainly Na^+^ and K^+^ (Thomas and Berkovic, 2014).

Several studies have shown that HCN channels are potentially involved in the pathogenesis of epilepsy (Tang et al., 2008; Baruscotti et al., 2010; Dibbens et al., 2010; DiFrancesco and DiFrancesco, 2015). Among the four different isoforms known (HCN1–4), each with different kinetic and voltage characteristics, isoforms 1, 2 and 4 are widely expressed in neurons, with a diverse distribution in brain areas. The role of HCN channels in neurons of the brain has been only partially clarified, but it is established that they contribute to the control of neuronal discharge and their dysfunction can lead to hyperexcitability and uncontrolled action potential firing, thus predisposing to seizures (Robinson and Siegelbaum, 2003; Biel et al., 2009; Benarroch, 2013; DiFrancesco and DiFrancesco, 2015).

Animal models lacking specific isoforms of the channel have shown different phenotypes. *HCN1* knockout leads to a significant increase of neuronal firing and hyperexcitability, without however causing spontaneous seizures (Huang et al., 2009; Santoro et al., 2010). HCN2 loss-of-function instead causes spontaneous generalized epilepsy, both in genetically modified animals (Ludwig et al., 2003) and in animals carrying a spontaneous truncating mutation (Chung et al., 2009).

In human patients, the evidence for *HCN* mutations promoting epilepsy is rapidly growing. So far, mutations in HCN2 have been found and characterized in patients with idiopathic generalized epilepsy (IGE; Tang et al., 2008; DiFrancesco et al., 2011), febrile seizures and genetic epilepsy with febrile seizures plus (Dibbens et al., 2010; Nakamura et al., 2013) and GGE (Li et al., 2018). Mutations in HCN1 have been described in infants affected by a severe form of progressive epileptic encephalopathy, with difficult control of seizures and poor prognosis (Nava et al., 2014) and very recently in a patient with GGE (Bonzanni et al., 2018).

HCN4 channels mediate pacemaker activity in the heart (Brown et al., 1979) and many mutations associated with cardiac arrhythmias have been characterized (Schulze-Bahr et al., 2003; Milanesi et al., 2006; Baruscotti et al., 2010; DiFrancesco, 2013, 2015).

Experimental data show that HCN4 is also highly expressed in the central nervous system (CNS), mainly in deep structures such as thalamic nuclei (Bender et al., 2001), hippocampus and spinal cord (Seo et al., 2015). The brain areas expressing HCN4 channels are critical for the development of seizures, and genetic alteration of these channels is potentially epileptogenic.

A recent report has indicated the presence of HCN4 loss-of-function mutations in generalized epilepsy patients (Becker et al., 2017). However, HCN4 variants were found in only a single affected individual, and changes of excitability following expression in neurons were not investigated.

Since HCN4 is highly expressed in cortical regions (including the hippocampus) specifically during infancy, and its expression rapidly declines with age (Brewster et al., 2007; Kanyshkova et al., 2009; Battefeld et al., 2012; Seo et al., 2015), HCN4 deficit may represent a potential mechanism for infantile forms of epilepsy.

Here, we report the identification of a mutation of HCN4 in two brothers affected by myoclonic epilepsy of infancy. By means of functional studies involving transfection of wild-type (WT) and mutant channels first into CHO cells and then into neurons, we show that this mutation determines a loss-of-function effect and, for the first time with HCN4 mutations, an increased neuronal excitability potentially contributing to the development of infantile epilepsy.

## Materials and Methods

### Patient Recruitment and Data Collection

We recruited patients with diagnosis of idiopathic generalized and partial epilepsy according to definition (Commission on Classification and Terminology of the International League Against Epilepsy, 1989). For all patients included, we collected information about gender, type of epilepsy (generalized, partial or undetermined) and inheritance of the disease, considered as sporadic (the patient is the only affected of the family) or familiar (at least one member of the proband’s family is affected by epilepsy with similar features). In order to identify a possible symptomatic etiology of seizures, clinical and instrumental data of patients with epilepsy were analyzed in detail. Structural causes, such as cerebrovascular disease, tumor or trauma, were investigated with 1 or 1.5 T brain MRI with proper sequences (T1, T1 with Gadolinium, T2/FLAIR, Inversion Recovery); biochemical and hematological tests were performed to exclude metabolic causes. Other seizure-provoking factors like antipsychotic or antidepressant therapy, alcohol or drug dependency, infection of the CNS were excluded. EEG was used to characterize features of epilepsy. Subjects with symptomatic epilepsy were excluded from recruitment. This study was carried out in accordance with the recommendations of the Italian Ministry of Health. The protocol was approved by the local Institutional Review Board of the Besta Institute and S. Gerardo Hospital (protocol number 1459). All subjects gave written informed consent in accordance with the Declaration of Helsinki. Upon acceptance of the informed consent, patients underwent a small blood withdrawal in EDTA anticoagulant for DNA extraction.

### DNA Extraction and Genetic Screening

Genomic DNA was prepared from peripheral-blood lymphocytes using standard procedures, as previously reported (DiFrancesco et al., 2014, 2015).

We used a TruSeq Custom Amplicon (Illumina), with a Studio Design software (Illumina Inc., San Diego, CA, USA) to customize a gene panel for the analysis of the genes coding for HCN ion channels and accessory proteins: *HCN1* (NM_021072; NP_066550), *HCN2* (NM_001194; NP_001185), *HCN4* (NM_005477; NP_005468), *APBA2* (NM_005503; NP_005494), *CAV3* (NM_033337; NP_203123), *MAGI2* (NM_012301; NP_036433), *FLNA* (NM_001110556; NP_001104026), *KCNE2* (NM_172201; NP_751951), *PEX5L* (NM_016559; NP_057643), *GRASP* (NM_181711; NP_859062).

Following the identification of the variant p.Arg550Cys (c.1648C>T) on *HCN4*, the DNA of both probands was analyzed with a more extended Next-Generation Sequencing (NGS) panel. This analysis was conducted in order to rule out any other possible causative genetic factor associated with the disease. We used a Nextera Rapid Capture method with Studio Design software (Illumina Inc., San Diego, CA, USA) using a customizing gene panel (see Table 1). The mean of coverage for this panel was 96%; the coverage of each gene is available on request. Obtained sequences were aligned to the reference genome (GRCh37/hg19) using MiSeq software. Data analysis was obtained using the following software: Illumina MiSeq Reporter vs. 2.4.60, Illumina Variant Studio vs. 2.2, Qiagen CLC Genomics Workbench vs. 7.0.

**Table 1:** List of genes analyzed in the customized panel.

DNA-binding protein	ARX, CHD2, EMX2, FOXG1, HESX1, MBD5, MECP2, MEF2C, TCF4, ZEB2
Enzyme	AFG3L2, ALDH7A1, CDKL5, CERS1, CSTB, CTSD, EMPM2A, GBA, KDM6A, KMT2D, MAGI2, NEU1, NHLRC1, PAFAHB1B, SMS, TPP1, EBE3A, WWOX
Ion channels and receptors	ATP1A2, CACNA1A, CHRNA2, CHRNB2, CLCN2, GABRB3, GABRG2, GRIN2B, HCN1, HCN2, HCN4, KCNC1, KCNE2, KCNQ2, KCNQ3, KCNT1, KCTD7, SCN1A, SCN1B, SCN2A, SCN8A
Structural proteins	ADGRG1, APBA2, CAV3, COL4A1, COL4A2, DCX, FLNA, GRASP, PCDH19, SLC2A1, SLC6A8, SLC9A6, TUBA1A, TUBB2B, TUBB3, TUBB8
Other	ARHGEF9, C10ORF2, CLN6, DEPDC5, GOSR2, NPC1, NPC2, PEX5L, SCARB2, SRPX2, STX1B, SYNGAP1, TBC1D24, VLDLR

Variants with MAF >1% reported in the dbSNP^1^, 1000 Genome^2^, EVS database^3^, ExAC database^4^ and gnomAD browser^5^ were considered benign variants and excluded from the report.

### Cell Culture

CHO cells (ATCC, cat# CCL-61, RRID:CVCL_0214) were cultured in F12 Nutrient mixture medium (Thermo Fisher Scientific) supplemented with 10% fetal bovine serum (FBS, Thermo Fisher Scientific), 1.5 g/L sodium bicarbonate, 2 mM L-glutamine, 100 U/ml penicillin and 100 g/ml streptomycin (Sigma-Aldrich).

Neonatal rat cortical neurons (NRCNs) were isolated as previously described (DiFrancesco et al., 2011). Sprague-Dowley rat pups from post-natal day 3 (Envigo, RRID:RGD_5508397) were euthanized by cervical dislocation, in accordance with the Italian and UE laws (D. Lgs n° 2014/26, 2010/63/UE) and following the approval of the Ethical Committee of the University of Milano and of the Italian Ministry of Health (protocol no. 1197/2015). Briefly, brains were removed and placed on ice in dissociation medium (in mmol/L: 134 Na-isethionic acid, 23 glucose, 15 HEPES, 10 kynurenic acid, 2 KCl, 4 MgCl_2_, 0.1 CaCl_2_, pH 7.2). The cerebral cortex was dissected from each brain hemisphere, minced with fine tweezers and digested in dissociation medium supplemented with 9.7 U/mL type XIV protease (Sigma-Aldrich) for 20 min at 37°C. Neurons were subsequently isolated mechanically using fire-polished Pasteur pipettes. 1 × 10^6^ cells were plated onto 35-mm poly-D-lysine-coated dishes in a mixture of dissociation medium and neurobasal medium, containing Neurobasal-A medium with 1× B27 supplement, 1 mM GlutaMAX I, 10 ng/mL human bFGF (all from Thermo Fisher Scientific), 50 U/ml penicillin and 50 g/ml streptomycin (Sigma-Aldrich), and let to adhere for 1 h at 37°C and 5% CO_2_. The medium was then replaced with fresh neurobasal medium and neurons were kept at 37°C and 5% CO_2_ until transfection.

Neurons selected for electrophysiological analysis were pyramidal neurons based on their morphology. Neurons with depolarized resting potential (more positive than −30 mV) or low input resistance (lower than 200 MΩ) were discarded.

Neonatal rat cardiomyocytes (NRVCs) were isolated from the same 3-day-old rats (Envigo), as previously reported (Avitabile et al., 2011). After heart removal, ventricles were chopped with fine tweezers in PBS and enzymatically digested by repeated digestions of 15 min at 37°C with collagenase I (136.8 U/ml, Whorthington) and pancreatine (0.6 mg/ml, Sigma-Aldrich) added to ADS solution (in mmol/L: 116.4 NaCl, 5.4 KCl, 1 NaH_2_PO_4_·H_2_O, 0.8 MgSO_4_·H_2_O, 5.5 glucose, 20 HEPES, pH 7.4). Cardiomyocytes were then plated onto 35-mm dishes and maintained in DMEM/M199 (Sigma-Aldrich), supplemented with 10% Horse Serum (Euroclone), 5% FBS (Thermo Fisher Scientific), L-glutamine 2 mmol/L and 1% Pen-Strep (Sigma-Aldrich) at 37°C and 5% CO_2_ until transfection.

### Plasmids and Transfection

The WT human (h) HCN4 cDNA sequence was cloned in pcDNA1.1 vector. Site-directed mutagenesis (QuikChange II Site-Directed Mutagenesis Kit, Agilent Technologies) was performed to introduce the R550C mutation using the following primers: F 5′-cgcccgacacccggcagtgcatccacgactactac-3′, R 5′-gtagtagtcgtggatgcactgccgggtgtcgggcg-3′. Automated DNA sequence analysis (BioFab Research, Italy) verified the mutation.

CHO cells plated on 35-mm dishes were transfected with 1.5 μg of either WT or R550C hHCN4 or with 0.75 μg of both using Fugene HD (Promega). The transfection of the same amount of WT and R550C constructs was used to mimic the heterozygous condition of the patients. Co-transfection of 0.3 μg pmaxGFP (Lonza) was used to select transfected cells for patch clamp experiments. Cortical neurons and ventricular myocytes were transfected the day after isolation using Lipofectamine 2000 (Thermo Fisher Scientific) following manufacturer instructions. For neuron transfection, we used 0.25 μg of WT or R550C hHCN4, or 0.12 μg of both, and 0.1 μg of pmaxGFP. For cardiomyocyte transfection, 1.2 μg of WT or R550C hHCN4, or 0.6 μg of both, and 0.3 μg of pmaxGFP were used.

### Electrophysiology

Electrophysiological analysis was performed 48 h after transfection using the patch-clamp technique in whole-cell configuration. CHO cells and NRVCs were dissociated with trypsin-EDTA (Sigma-Aldrich) and plated at low density on 35-mm dishes, in order to record hHCN4 current from single cells. Ventricular myocytes were kept at 36 ± 1°C while CHO cells and cortical neurons were kept at room temperature. CHO cells and NRVCs were kept in Tyrode solution containing (in mmol/L): 140 NaCl, 5.4 KCl, 1.8 CaCl_2_, 1 MgCl_2_, 5.5 D-glucose, 5 Hepes-NaOH; pH 7.4. NRCNs were kept in a physiological extracellular solution containing (in mmol/L): 129 NaCl, 35 glucose, 10 Hepes, 3 KCl, 1.8 MgSO_4_, 1.6 CaCl_2_, 1.25 NaH_2_PO_4_; pH 7.4. In CHO cells, the hHCN4 current was recorded using a high-K solution containing (in mmol/L): 110 NaCl, 30 KCl, 5 Hepes-NaOH, 1.8 CaCl_2_, 0.5 MgCl_2_, 1 BaCl_2_, 2 MnCl_2_, pH 7.4. In NRVCs, the hHCN4 current was dissected by adding 1 BaCl_2_, 2 MnCl_2_ to Tyrode solution.

For recordings in CHO cells and NRVCs, patch-clamp pipettes had a resistance of 4–7 MΩ when filled with the intracellular-like solution containing (in mmol/L): 130 KCl, 10 NaCl, 5 EGTA-KOH, 0.5 MgCl_2_, 2 ATP (Na-salt), 5 creatine phosphate, 0.1 GTP, 10 Hepes-KOH; pH 7.2. For recordings in NRCNs, pipettes had a resistance of 7–10 MΩ and were filled with an intracellular-like solution containing (in mmol/L): 120 K-gluconate, 20 P-creatine, 15 KCl, 10 Hepes, 2 MgCl_2_, 2 ATP, 0.2 GTP, 0.2 EGTA, 0.1 leupeptin; pH 7.2.

hHCN4 currents were recorded from GFP-expressing cells in voltage-clamp mode, applying voltage steps in 20 mV increments to the range −35/−135 mV or to the range −35/−125 mV, followed by a fully-activating step to −135 mV or −125 mV for CHO cells and cardiac myocytes, respectively. In cortical neurons, hHCN4 current was activated by hyperpolarizing steps in 20 mV increments to the range −35/−115 mV. At each voltage, steps were long enough to reach steady state of current activation. The holding potential (hp) was set to −30 mV in all experiments. Activation curves were obtained from tail currents (for CHO cells and cardiomyocytes) or by calculating the conductance at each voltage step and normalizing it to maximum conductance (for cortical neurons) and were fitted to the Boltzmann equation:

$$y=1/\left(1+exp\left((V-V_{1/2})/s\right)\right)$$

where V is voltage, y the fractional activation, V_1/2_ the half-activation voltage, and s the inverse-slope factor. hHCN4 current density was obtained from current amplitude at each potential normalized to cell capacitance. Cell capacitance was calculated by integrating capacitive currents elicited by a 10-mV voltage step from −35 mV to −45 mV. Time constants were obtained by fitting activation and deactivation current traces with a single exponential function. Action potential firing of cortical neurons was recorded in current-clamp mode by holding membrane potential at −70 mV for 3 s and applying depolarizing current steps in 10 pA increments.

### Experimental Design and Statistical Analysis

Data were analyzed with Clampfit (Axon) and Origin Pro 9 (Origin Lab). Activation curves were compared by analyzing the V_1/2_ using one-way ANOVA followed by Fisher’s LSD *post hoc* test; significance level was set to *p* = 0.05. Data outliers were excluded using Tukey’s method. Data were collected from at least three different transfection experiments or primary cultures.

## Results

### Identification of the HCN4 p.Arg550Cys Variant in Familiar Epilepsy

For the present study, we recruited 88 patients affected by idiopathic epilepsy, which was classified as generalized, partial or undetermined (see Table 2 for details). The disease had properties compatible with familial inheritance in 38 cases and was apparently sporadic in 50.

**Table 2:** Characteristics of epileptic patients.

Gender	Number	%
F	37	42
M	51	58
**Type of epilepsy**		
Generalized	73	83
Partial	10	11
Undetermined	5	6
**Inheritance**		
Familiar	38	43
Sporadic	50	57

**Figure 1:**
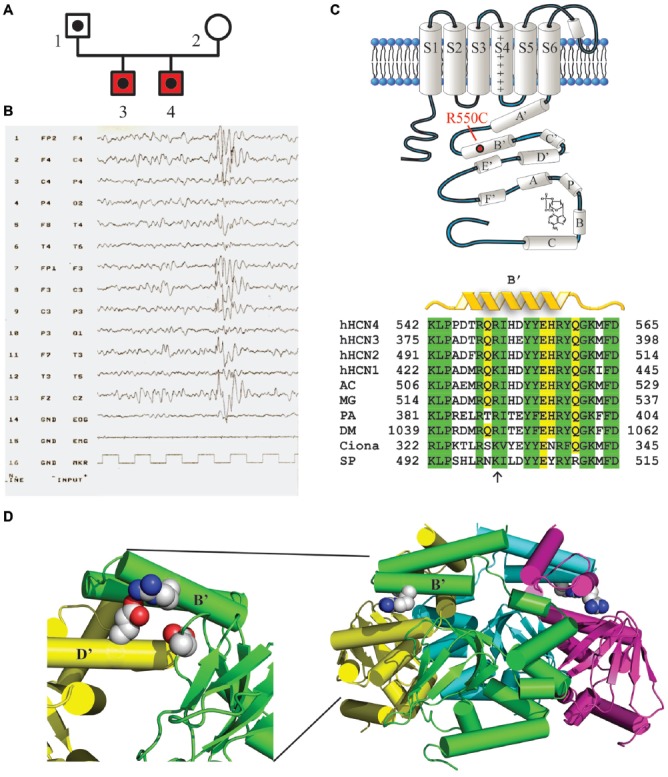
Mutation R550C found in the HCN4 C-linker region of epileptic siblings. **(A)** Family pedigree. Dotted symbols represent individuals carrying the HCN4 R550C mutation in heterozygosis (probands and their father); red symbols indicate reported diagnosis of benign myoclonic epilepsy of infancy. **(B)** EEG of patient 3 showing normal background, with generalized epileptiform activity prevalent in the anterior regions. (**C**, top) Schematic representation of one HCN4 channel subunit showing the six transmembrane domains (S1–S6) and the intracellular N- and C-termini. The C-terminus includes the C-linker, comprising six α-helices (A′ to F′), and the cyclic nucleotide-binding domain (CNBD, A to C), as indicated. The approximate position of the mutation R550C in the B’ helix is also shown. (**C**, bottom) Sequence alignment of the B’ helix of the four human HCN channel isoforms (hHCN4 NP_005468.1, hHCN1 NP_066550.2, hHCN2 NP_001185.3, hHCN3 NP_065948.1) and of homologous channel regions in other species. Note that at position hHCN4:R550 a positively charged amino acid (arrow) is conserved in all species indicated. AC, *Anolis carolinensis* (XP_008119223.1); MG, *Meleagris gallopavo* (XP_010715467.1); PA, *Panulirus argus* (AAQ16311.1); DM, *Drosophila melanogaster* (NP_001137667.1); Ciona, *Ciona intestinalis* (AFB83348.1); SP, *Strongylocentrotus purpuratus* (NP_999729.1). (**D**, right) Ribbon 3D representation of the tetrameric arrangement of hHCN4 C-termini, comprising C-linkers and CNBDs, based on X-ray crystallographic data (Xu et al., 2010; PDB ID: 3OTF). R550 residues in B’ α-helices are drawn as space-filling plots. (**D**, left) Expanded and rotated view showing residues R550 (green B’ helix), E580 (yellow D’ helix) and D620 (green β-roll), which may act as salt-bridges (Craven and Zagotta, 2004).

The genetic screening of patients with TruSeq technology identified the HCN4 variant p.Arg550Cys (c.1648C>T, rs150691273) in two brothers (Figure 1A). The residue Arg550 is located in the B’ helix of the C-linker (Figure 1C, top), as also visible in the 3D structure of the C-terminus (Figure 1D, right) reconstructed from X-ray data (Xu et al., 2010). A positively charged amino acid is highly conserved among different species (Figure 1C, bottom), suggesting a significant functional role. The p.Arg550Cys mutation determines a charge variation, from the positively charged residue arginine to the polar amino acid cysteine. Biophysical studies have shown that the homologous residue in mHCN2 channels (K472) links through salt bridges with two other residues (E502 and D542), forming inter- and intra-subunit interactions which help stabilize the tetrameric arrangement of the C-terminal domain and can, when disrupted, modify channel kinetics (Craven and Zagotta, 2004). The expanded view of Figure 1D (left) shows the positions of the homologous hHCN4 residues (R550, E580 and D620). Interatomic distances are similar to those found in mHCN2 (data not shown), suggesting that salt bridges are functionally important also in human HCN4 channels.

We extended the genetic screening within the family of the two probands and found that this variant had been inherited by their father, for whom we lack anamnestic data relative to his youth. We can therefore neither exclude nor confirm the presence of infantile seizures in this subject. In order to rule out any further genetic abnormalities putatively responsible for the medical case history, both probands underwent additional NGS analysis using a Nextera approach, aimed to screen a large set of genes known to be involved in the pathogenesis of epilepsy (see Table 1 for a complete list). This additional panel did not identify any other genetic variants of potential interest.

### Case Description

The two brothers investigated here were born from a physiological pregnancy, by non-consanguineous parents. Their phenotype and the evolution of their clinical histories are similar in many ways. They both presented the first seizures before the first year of age, characterized by loss of consciousness and myoclonus of the four limbs, with occasional drop to the ground. Medical history was negative for febrile seizures, traumatic brain injury or infections. EEG analysis showed normal background, with generalized epileptiform activity prevalent in the anterior regions, associated with myoclonus induced by acoustic stimulus, with negative intermittent photic stimulation (Figure 1B, patient 3). Neuroradiological examinations were negative. Based on clinical and EEG findings, they were diagnosed with benign myoclonic epilepsy of infancy and treatment with valproate was started with full remission of seizures, which terminated at around $2\frac{1}{2}$ years of age for both brothers. Anti-epileptic treatment was interrupted when the brothers were 4 years of age, without reporting any additional seizures. Both patients showed a modest delay in language development, recovered by logopedics. In patient 3 only, follow-up EEG remained positive for interictal epileptic activity until the age of 12. For patient 4 a formal cognitive assessment showed a subclinical mental disability (IQ 76).

### The R550C Mutation Causes a Negative Shift of the HCN4 Channel Activation Curve

To verify if the R550C mutation modifies functional properties of HCN4 channels, we transfected CHO cells with WT, homozygous mutant (R550C) or heterozygous WT/mutant (WT/R550C) HCN4 channels and performed patch-clamp analysis.

**Figure 2:**
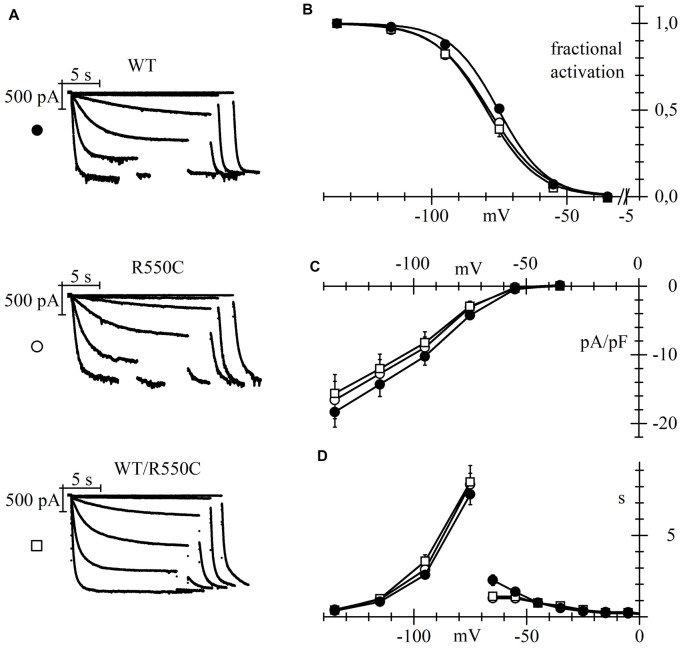
The R550C mutation causes a shift of the activation voltage-dependence of HCN4 expressed in CHO cells. **(A)** Representative hHCN4 current traces recorded during a two-step activation protocol (test range −35/−135 mV, holding potential (hp) = −30 mV) in CHO cells transfected with either wild-type (WT), R550C or WT/R550C hHCN4, as indicated. **(B)** Plot of the mean activation curves of hHCN4 currents (WT, filled circles; R550C, open circles; WT/R550C, open squares). Mutated channels had a slightly, but statistically significant, more negative range of activation than WT channels. V_1/2_ and s values from Boltzmann curve fitting were (mV): WT, −74.9 ± 1.0, 8.52 ± 0.38 (*n* = 33); R550C, −79.1 ± 1.0, 9.41 ± 0.47 (*n* = 29); WT/R550C, −79.5 ± 1.7, 8.50 ± 0.42 (*n* = 20). **(C)** I/V density curves. Current densities at −135 mV were (pA/pF): WT, −18.3 ± 2.2 (*n* = 24); R550C, −16.6 ± 2.7 (*n* = 22); WT/R550C, −15.6 ± 2.8 (*n* = 17). **(D)** Mean time constants of activation (negative to −75 mV) and deactivation (positive to −65 mV). In **(C,D)** no significant differences were found among the three channel types, except deactivation time constant at −65. Meaning of symbols as in **(A,B)**.

Representative traces of HCN4 currents recorded from CHO cells expressing the three different channels, and plots of the mean activation curves thus obtained, are shown in Figures 2A,B.

The voltage dependence of activation of both homozygous mutant and heterozygous WT/mutant channels was shifted by about 4–5 mV towards more negative voltages relative to WT channels.

Statistical comparison of the activation curves showed that both R550C and WT/R550C curves were significantly different from the WT curve (*F*_(2,79)_ = 4.917, *p* = 0.010). V_1/2_ values of R550C and WT/R550C were similar (*t* = −0.24, *p* = 0.811) and significantly different from WT (*t* = −2.65, *p* = 0.009; *t* = −2.63, *p* = 0.010 for R550C and WT/R550C, respectively).

On the other hand, current densities did not change significantly (Figure 2C; *F*_(2,60)_ = 0.290, *p* = 0.750, one-way ANOVA at −135 mV) nor did time constants of activation and deactivation (Figure 2D), with the exception of the deactivation time constant at −35 mV (*F*_(2,23)_ = 7.519, *p* = 0.003).

We also verified if the R550C mutation had any effect on the cAMP-induced channel activation (DiFrancesco and Tortora, 1991) by comparing currents recorded in the presence and in the absence of cAMP in the recording pipette.

**Figure 3:**
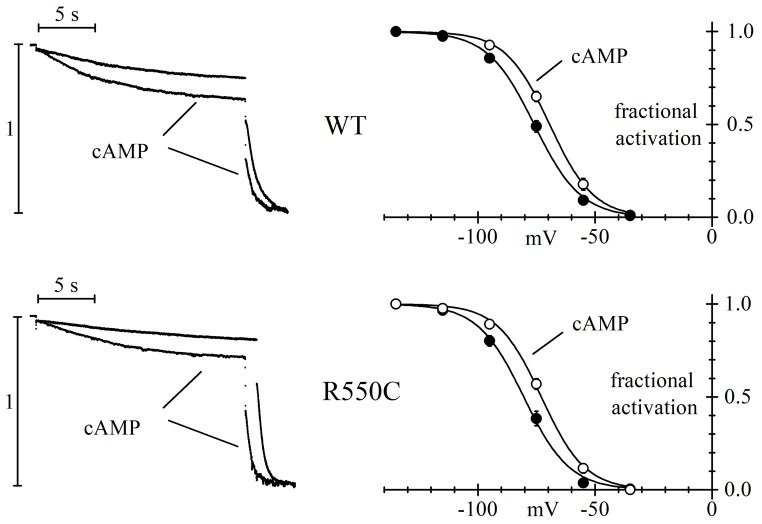
The R550C mutation does not affect the cAMP dependence of HCN4 channels. Representative current traces recorded during steps to −75/−125 mV from a hp of −35 mV (left) and mean activation curves (right) in CHO cells expressing WT (top) or R550C mutated channels (bottom). Records and activation curves labeled “cAMP” were obtained with patch pipettes filled with the standard intracellular-like solution to which 10 μm cAMP was added. Sample traces on the left are normalized to maximal amplitude at −125 mV. cAMP shifted the hHCN4 channel activation curve by 7.5 and 7.2 mV in WT and R550C-expressing cells, respectively. V_1/2_ and s values from Boltzmann fitting were (mV): WT, −76.7 ± 1.4, 9.41 ± 0.49 (*n* = 16); WT+cAMP, −69.2 ± 1.2, 8.80 ± 0.78 (*n* = 16); R550C, −80.1 ± 1.6, 8.72 ± 0.59 (*n* = 14); R550C+cAMP, −72.9 ± 1.1, 9.02 ± 0.51 (*n* = 13).

The left panels of Figure 3 show sample current traces recorded from cells expressing either WT (top) or R550C mutant channels (bottom) during a two-step voltage clamp to −75/−125 mV (mid/full activation range).

In both cases, traces recorded in the presence of 10 μm cAMP in the whole-cell pipette showed, after normalization to maximal current, an increase at −75 mV and a decrease at −125 mV, indicating a shift of the activation curve to more positive voltages in the presence of cAMP. In the right panels, a more complete analysis shows that the mean activation curves of WT and R550C channels, as fitted to the Boltzmann equation, cause statistically significant V_1/2_ shifts of 7.5 and 7.2 mV, respectively (cAMP vs. control: WT, *F*_(2,178)_ = 27.83, *p* < 0.0001; R550C, *F*_(2,150)_ = 33.32, *p* < 0.0001) These data indicate that the R550C mutation does not modify the cAMP-dependence of channel activation.

### The HCN4 R550C Mutation Increases Neuronal Excitability by a Loss-of-Function Effect

**Figure 4:**
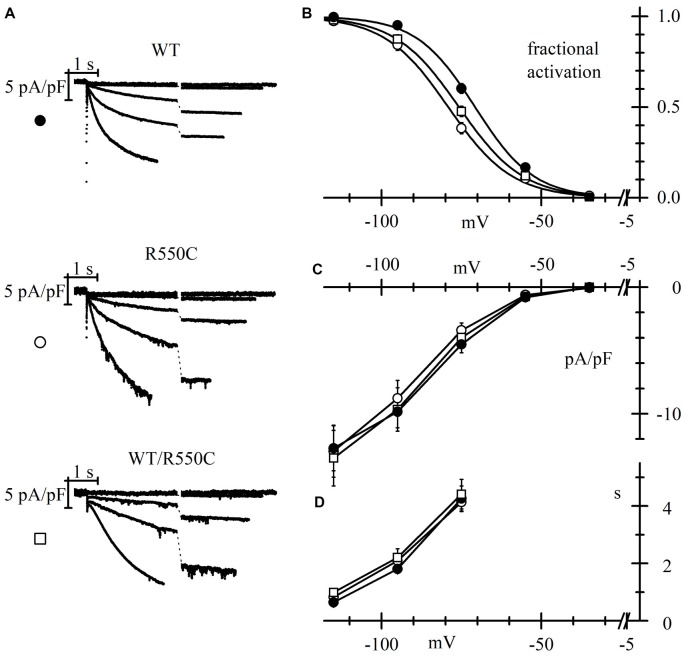
The R550C mutation causes a shift of HCN4 activation curve when expressed in rat neonatal cortical neurons. **(A)** Representative hHCN4 current traces recorded during a single step protocol (test range −35/−115 mV, hp = −30 mV) in cortical neurons transfected with either WT, R550C or WT/R550C hHCN4, as indicated. Breaks in the current traces correspond to a 8.5 s duration. **(B)** Plot of the mean hHCN4 activation curves (meaning of symbols as in Figure 2). Mutant channels have activation curves shifted by about 5–8 mV in the negative direction relative to WT channels. V_1/2_ and s values from Boltzmann fitting were (mV): WT, −71.1 ± 1.1, 8.4 ± 0.3 (*n* = 21); R550C, −78.8 ± 1.1, 9.2 ± 0.5 (*n* = 18); WT/R550C, −75.8 ± 1.2, 9.8 ± 0.6 (*n* = 12). **(C)** I/V density curves. Current densities at −115 mV were (pA/pF): WT, −12.3 ± 1.5 (*n* = 20); R550C, −12.7 ± 1.8 (*n* = 19); WT/R550C, −13.1 ± 2.2 (*n* = 19). **(D)** Mean time constants of activation. In **(C,D)** no significant differences were found among the three channel types.

In order to evaluate if HCN4 channels carrying the R550C mutation are able to modify neuronal excitability, we transfected NRCNs with either WT, R550C or WT/R550C HCN4 channels, and recorded currents in patch-clamp experiments (Figure 4).

As expected from the properties of HCN channels, transfection of all channel types resulted in a depolarization of the resting membrane potential and a strong decrease of input resistance when comparing with untransfected channels (see Table 3).

**Table 3:** Electrophysiological properties of untransfected and transfected neurons.

	Untransfected	WT	R550C	WT/R550C
Resting potential	−52.7 ± 3.9	−39.3 ± 1.5*	−42.2 ± 1.3*	−42.0 ± 1.1*
(mV)	*n* = 8	*n* = 27	*n* = 32	*n* = 25
Input resistance at −70 mV	1814.3 ± 230.7	522.5 ± 54.5*	744.8 ± 89.6*^§^	910.1 ± 80.7*^§^
(MΩ)	*n* = 8	*n* = 27	*n* = 32	*n* = 25

*p < 0.05 vs. untransfected; ^§^*p* < 0.05 vs. WT. All tests by one-way ANOVA and Fisher LSD *post hoc* test.

Expression in neurons revealed mutant channel properties similar to those observed in CHO-transfected cells. The V_1/2_ of activation curves of both homozygous R550C and heterozygous WT/R550C channels were similar (*t* = 1.725, *p* = 0.091) and shifted to more negative voltages by 7.7 (R550C) and 4.7 mV (WT/R550C) relative to the WT activation curve (*t* = −5.13, *p* = 5.03e-6; *t* = −2.78, *p* = 0.007, respectively).

Also as in CHO cells, the R550C mutation did not affect mean current densities (Figure 4C; *F*_(2,55)_ = 0.04, *p* = 0.96, one-way ANOVA at −135 mV). We also did not find significant changes in the voltage dependence of the time constants of activation (Figure 4D).

The negative shift of the activation curve indicates a loss of function of the channel. Since HCN channels are open at physiological resting membrane potentials of neurons (Pape, 1996; Doan and Kunze, 1999; Lupica et al., 2001; Meuth et al., 2006; Nolan et al., 2007; Biel et al., 2009), a loss of function and the consequent, reduced HCN4 current contribution may affect input membrane resistance as well as resting membrane potential (Dyhrfjeld-Johnsen et al., 2009) and thus impact cell excitability.

Indeed, in agreement with the evidence of a loss of function, transfection of mutant channels led to a significant increase of neuronal input resistance relative to WT channels, as shown in Table 3.

In order to address this issue, we sought to compare membrane excitability of neurons transfected with either WT, R550C or WT/R500C HCN4. We injected depolarizing current steps of progressively larger amplitude in neurons (previously stabilized to a resting potential of −70 mV) and recorded by patch-clamp their electrical activity.

**Figure 5:**
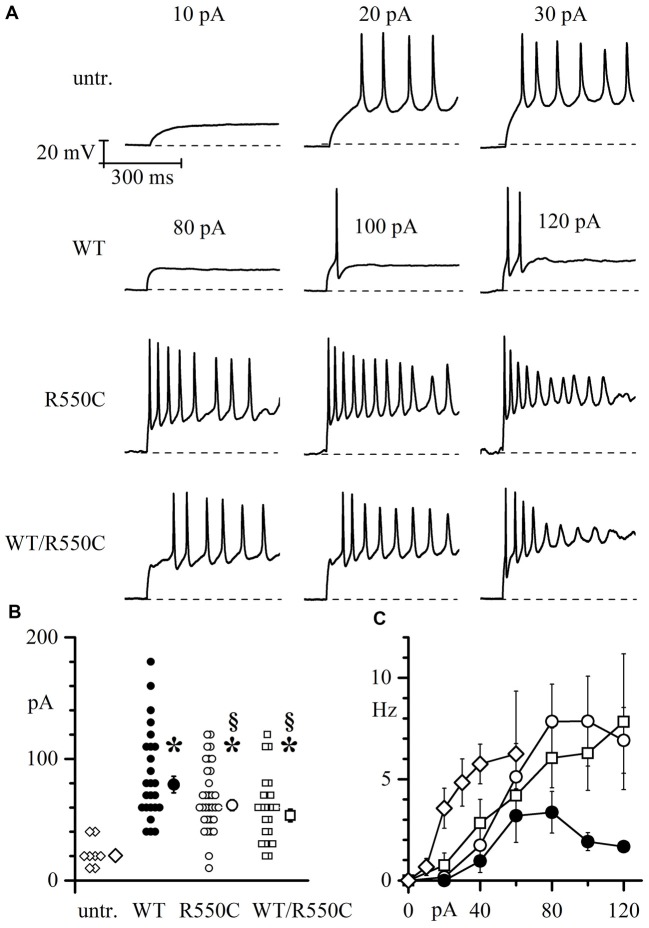
Membrane excitability of neonatal rat cortical neurons (NRCNs) increases in cells expressing mutant vs. WT channels. **(A)** The top three panels show representative voltage traces recorded upon injection of 10, 20 and 30 pA current steps applied from cells held at −70 mV (dashed lines) in untransfected cortical neurons. The remaining panels show traces recorded upon injection of 80, 100 and 120 pA current steps in cortical neurons transfected with either WT, R550C or WT/R550C hHCN4, as indicated. Time and voltage scales apply to all panels. **(B)** Plot of the current necessary to trigger the first action potential (current threshold) in untransfected (open diamonds), WT (filled circles), R550C (open circles) and WT/R550C-transfected neurons (open squares). Shown are all recorded data and mean ± SEM values (in pA: untransfected: 22.5 ± 4.12, *n* = 8; WT: 86.8 ± 7.5, *n* = 25; R550C: 68.1 ± 4.9, *n* = 32; WT/R550C: 58.8 ± 5.6, *n* = 24; *p < 0.05 relative to untransfected; ^§^*p* < 0.05 relative to WT). **(C)** Plot of the mean firing rate, measured during the first 500 ms, as a function of the current step injected in untransfected neurons or neurons transfected with WT, R550C or WT/R550C channels (symbols as in **B**).

Representative traces in Figure 5A show that a current step of 20 pA was sufficient to elicit firing in one untransfected neuron (top panels), while in another neuron expressing WT channels (second row of panels labeled “WT”), cell excitability was much lower and firing of only a few action potentials could only be evoked by the largest current injections applied (100–120 pA). These data confirm similar findings previously obtained with HCN2 channels (DiFrancesco et al., 2011) indicating that transfection of the WT isoform strongly reduces native neuronal firing and represent further evidence for a functional role of HCN channels in modulating excitability.

On the other hand, when neurons were transfected with R550C or WT/R550C mutant HCN4 channels (third and fourth rows in Figure 5A), a higher level of cell excitability was observed, and firing of action potential trains could be elicited with current steps of 80 pA and less.

As shown in the more complete analysis of Figure 5B, expression of HCN channels clearly led to a large increase of the firing threshold relative to untransfected neurons (*F*_(2,87)_ = 10.149 *p* = 8.77*10^−6^; untr. vs. WT *t* = −5.28, *p* = 9.556*10^−7^; untr. vs. R550C *t* = −3.85, *p* = 2.25*10^−4^; untr. vs. WT/R550C *t* = −2.96, *p* = 0.004). Furthermore, expression of mutant channels caused neurons to fire on average at a significantly lower current threshold than WT-expressing neurons (WT vs. R550C: *t* = −2.336, *p* = 0.022; WT vs. WT/R550: *t* = −3.277, *p* = 0.001; R550C vs. WT/R550C: *t* = −1.116, *p* = 0.250), and the mean firing rate of R550C and WT/R550C-transfected neurons, as measured in the first 500 ms of current injection, was higher compared to WT-transfected neurons (Figure 5C).

### The R550C Mutation Alters the Properties of HCN4 Channels Expressed in Neonatal Rat Ventricular Cardiomyocytes

It is well established that HCN4 channels are strongly expressed in cardiac cells where they contribute essentially to generation of cardiac pacemaker activity and control of cardiac rate (DiFrancesco, 1993, 2010a,b; Baruscotti et al., 2010; DiFrancesco and Noble, 2012; Wahl-Schott et al., 2014). We sought to investigate whether the modifications induced by the R550C mutation are also maintained when channels are transfected into cardiac myocytes.

We transfected either WT, R550C or WT/R550C HCN4 channels in neonatal rat ventricular cardiomyocytes and performed patch-clamp experiments to analyze their biophysical properties. We choose neonatal rat ventricular myocytes since although they express an endogenous I_f_ current (Avitabile et al., 2011) which is carried mostly by HCN2 channels (Shi et al., 1999), this is much smaller than and does not interfere with the transfected HCN4 current.

The modifications induced by the R550C mutation on the properties of HCN4 expressed in cardiomyocytes were similar to those observed in CHO cells and in cortical neurons.

**Figure 6:**
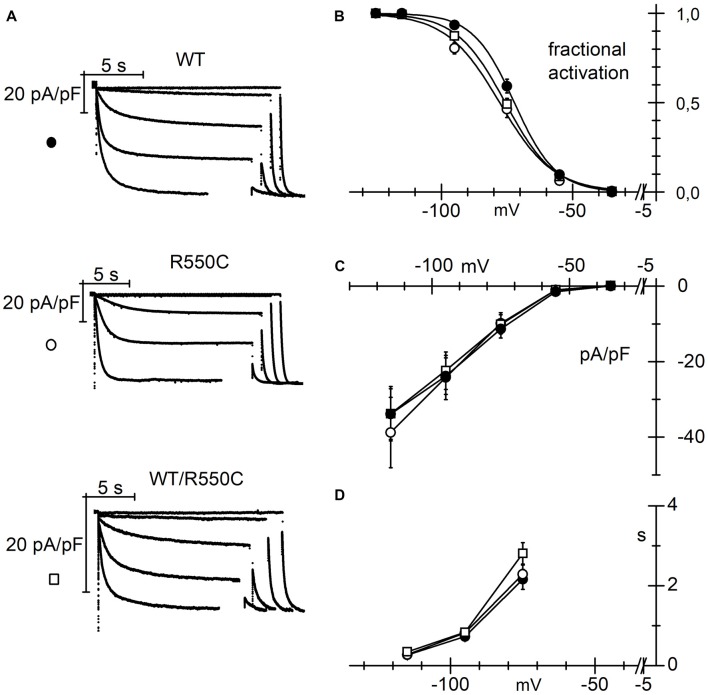
The R550C mutation causes a negative shift of the HCN4 activation curve when expressed in cardiac myocytes. **(A)** Representative hHCN4 current traces recorded during a two-step activation protocol (test range −35/−125 mV, hp = −30 mV, fully activating voltage = −125 mV) in ventricular myocytes transfected with either WT, R550C or WT/R550C hHCN4, as indicated. **(B)** Plot of the mean activation curves of hHCN4 currents. Mutated channels have activation curves shifted by −3.7 to −6 mV relative to WT. V_1/2_ and s values from Boltzmann fitting were (mV): WT, −72.4 ± 1.2, 7.0 ± 0.4 (*n* = 21); R550C, −78.4 ± 1.3, 9.7 ± 0.7 (*n* = 15); WT/R550C, −76.1 ± 1.0, 8.7 ± 0.6 (*n* = 21). **(C)** I/V density curves. Current densities at −125 mV were (pA/pF): WT, −33.9 ± 6.7 (*n* = 19); R550C, −38.8 ± 9.3 (*n* = 12); WT/R550C, −33.8 ± 7.2 (*n* = 17). **(D)** Mean time constants of activation. In **(C,D)** no significant differences were found among the three channel types.

Measurement of activation curves with the protocols illustrated in Figure 6A showed that mutant channels activate at slightly but significantly more negative voltages than WT channels (Figure 6B; *F*_(2,54)_ = 6.419, *p* = 0.003, one-way ANOVA at −125 mV).

Activation curves of R550C and WT/R550C channels had V_1/2_ values not significantly different from each other (*t* = 1.32, *p* = 0.192) but underwent statistically significant shifts of about 6 and 3.7 mV relative to the WT curve (*t* = −3.47, *p* = 0.001; *t* = −2.36, *p* = 0.021, respectively). No differences were found in the current densities of mutant vs. WT channels (Figure 6C; *F*_(2,45)_ = 0.12, *p* = 0.89, one-way ANOVA), nor in the time constants of activation (Figure 6D).

Negative shifts of the activation range of HCN4 channels caused by specific channel mutations have often been associated with symptomatic or asymptomatic bradycardia (Schulze-Bahr et al., 2003; Milanesi et al., 2006; DiFrancesco, 2013). Although neither patient presented overt cardiac abnormalities, in childhood or adulthood, we decided to analyze the cardiac performance of the family members carrying the HCN4 R550C mutation. Both patients and their father therefore underwent a detailed cardiac characterization with electrocardiograms (ECG), 24-h Holter ECG and transthoracic echocardiogram. No structural abnormalities were found in any of the subjects.

ECGs taken at rest indicated that both probands had rates of 59 bpm, values which are just below the upper limit of bradycardic rates. Interestingly, the father (individual 1), also carrying the heterozygous R550C mutation, had a resting rate only slightly higher (62 bpm). These observations suggest that the slight negative shift of the activation curve associated with the R550C mutation is compatible with the moderately low heart rates of individuals carrying the mutation.

Taken together our results show that the R550C mutation, identified in two epileptic brothers with the same epileptic phenotype, similarly modifies the HCN4 channel biophysical properties in CHO cells, in neurons and in cardiac myocytes. In particular, in neurons this mutation produces an increased membrane excitability, compatible with a potential predisposition to epilepsy.

## Discussion

With the exception of few epileptic syndromes, such as the Dravet syndrome, the genetic causes of epilepsies are generally poorly understood. HCN ion channels are now emerging as potential key players in human epilepsy (Reid et al., 2012; Shah et al., 2013; DiFrancesco and DiFrancesco, 2015), and HCN channel mutations have already been identified in patients and associated with epilepsy (Dibbens et al., 2010; DiFrancesco et al., 2011; Nakamura et al., 2013; Nava et al., 2014).

We have identified and characterized an *HCN4* variant (R550C) in two brothers affected by benign myoclonic epilepsy of infancy. Electrophysiological characterization showed that this mutation induces a leftward shift in the voltage dependence of HCN4 activation both in homo and heterozygosis, according to a dominant effect of the mutation. Compared to WT, fewer mutated channels are open at rest, leading to reduced I_h_ and higher input resistance, changes normally associated with increased membrane excitability (Dyhrfjeld-Johnsen et al., 2009). Our functional studies indeed show that neurons expressing mutant channels present lower current thresholds to firing and higher firing rates. These alterations are consistent with a predisposition to develop the epileptic phenotype (DiFrancesco and DiFrancesco, 2015).

Even if our analysis of the patients did not reveal, other than the R550C mutation, significant variants in any of the epilepsy-related genes screened, we cannot exclude further contribution of mutations in other genes either unrelated or rarely associated with epilepsy. Furthermore, we cannot rule out the presence of mutations in the large portion of the non-coding genome (e.g., long non-coding RNA, miRNA) which, influencing gene expression, can potentially contribute to the penetrance of a disease (Crino, 2007; Shao and Chen, 2017).

Within the general population, the *HCN4* R550C variant is rare, with a calculated frequency of 8.122e-6^6^. The HCN4 variant we found is characterized by a point mutation in the B’ helix of the channel C-linker, whereby a positively charged arginine is replaced by the polar amino acid cysteine. A positively charged residue (arginine or lysine) is highly conserved in the same position in all HCN isoforms and across species, suggesting a highly relevant functional role.

Interestingly, it has been previously reported that the corresponding residue of Arg-550 in the *HCN2* isoform (Lys-472) is one of the three fundamental residues generating salt bridges, responsible for both inter- and intra-subunit interaction in the tetrameric structure of the channel. Replacement of any of these charged residues with residues bearing a charge of opposite sign cause alterations of the channel kinetics (Craven and Zagotta, 2004), thus confirming the importance of the conserved positive charge in this position. Our data show that the R550C mutation alters the channel kinetics by causing a shift of the activation curve voltage dependence to more negative voltages, thus reducing the channel contribution to keep a low input resistance.

The genetic causes of benign myoclonic epilepsy of infancy are known only in a small proportion of patients. Most mutations reported so far involve *KCNQ2/3* (Castaldo et al., 2002) and *SCN1A* genes (Gambardella and Marini, 2009), although in the majority of cases, functional studies supporting evidence of causative links are lacking.

The patients carrying the *HCN4* R550C mutation described here are affected by a mild form of infantile generalized epilepsy, with good response to pharmacological treatment, benign evolution and absence of sequelae in adulthood. This phenotype is clearly milder than the pharmacoresistant generalized epilepsy with tonic-clonic seizures with autosomal recessive inheritance associated with the HCN2 mutation (E515K) we previously reported (DiFrancesco et al., 2011). In the case of the HCN2 mutation, a serious phenotype was associated with a nearly complete loss-of-function of the channel contribution to activity. In the present work, on the other hand, we have found a less dramatic loss-of-function change of HCN4 contribution, in association with an epileptic phenotype which is milder and restricted to infancy.

The data we show in Figure 5, although in non-physiological conditions, represent the first experimental evidence that dysfunctional HCN4 channels can induce change in neuronal excitability.

HCN4 channels are widely expressed in the CNS, where they are mainly concentrated in the thalamus, especially in the relay nuclei, habenular complex and basal ganglia (Moosmang et al., 1999; Santoro et al., 2000; Notomi and Shigemoto, 2004; He et al., 2014). HCN4 channels have been most thoroughly characterized for their role in cardiac pacemaking (Baruscotti et al., 2011; DiFrancesco and Noble, 2012) and several mutations have been associated with alterations of cardiac rhythm (Milanesi et al., 2006; DiFrancesco, 2013; Baruscotti et al., 2017). Full understanding of the specific functions of HCN4 in neurons is however still lacking, and the impact of dysfunctional mutant channels has yet to be determined.

Nonetheless there are several indications that HCN4 channels have a role in neuronal excitability. For example, an association between HCN4 expression and high-frequency action potential discharge has been reported in rat hippocampal interneurons (Hughes et al., 2013).

Importantly, HCN4 expression in cortical regions is high at early stages of life and declines rapidly at later stages, suggesting a potential role of HCN4 dysfunctional behavior more specifically in infantile forms of epilepsy.

Studies focusing on the role of HCN channel during brain development have shown that HCN4 is highly expressed in hippocampal CA1 neurons during the first postnatal period (accounting for 30%–40% of the I_h_ current), whereas its expression strongly decreases in adulthood (contributing only for the 4%–8% of the total current).

Age-dependent adaptation of HCN4 expression is also suggested by the fact that during brain development the I_h_ properties change in ways compatible with HCN4 reduced expression: the I_h_ current becomes less sensitive to cAMP, acquires a faster activation time constant and its activation range shifts to more negative voltages, as expected with a decreased HCN4 contribution (Surges et al., 2006; Brewster et al., 2007).

Similar results on HCN4 expression during development were obtained in different mouse hippocampal regions (Seo et al., 2015). In thalamocortical neurons, too, HCN4 expression contributes to the I_h_ current at early postnatal stages, while at later stages the expression of HCN1 and HCN2 isoforms becomes prominent (Kanyshkova et al., 2009). More recently, a high perinatal expression of HCN4 and its strong decrease later in life were reported in the developing neocortex (Battefeld et al., 2012). Changes in developmental expression of various HCN subunits, and in particular of HCN4 and HCN1, is further supported by the compensation through HCN4 upregulation observed in the neocortex of HCN1 knockout mice (Stoenica et al., 2013).

The above evidence suggests that most of the neuronal phenotypic effects of HCN4 alterations may be restricted within a limited period of time, pointing to a possible transient effect of altered HCN4 on CNS function. We can thus speculate that the HCN4 R550C mutation affects neuronal excitability only in the early years of life, while the WT HCN1 and/or HCN2 isoforms can subsequently compensate to re-establish normal physiological conditions. This hypothesis is in agreement with the phenotype of the patients described here, who presented epileptic seizures only for the first $2\frac{1}{2}$ years of life.

On the other hand, the contribution of HCN4 is fundamental for maintaining normal heart rhythm also in adulthood, and since HCN channel properties are known to vary in different cell types (Qu et al., 2002), we sought to verify whether the R550C mutation might also have an effect when expressed in cardiac myocytes. Our results in rat neonatal cardiomyocytes show that the mutation produces a modest negative shift in the activation curve of the channel (Figure 6B). While our patients (individuals 3 and 4 in Figure 1A) did not present a symptomatic, overt cardiac phenotype, their resting heart rate was in both cases 59 bpm, a value which can be considered as borderline bradycardia, and their father had an only slightly higher rate of 62 bpm. These data are coherent with the known correlation between HCN4 loss-of-function mutations and bradycardia (DiFrancesco, 2013, 2015; Verkerk and Wilders, 2015).

It is however important to note that, to our knowledge, none of the patients already described with cardiac arrhythmias due to *HCN4* mutations has been reported to also present an epileptic phenotype, suggesting that the HCN4 R550C mutation reported here is likely to be a contributing factor requiring other mechanisms or a still unidentified genetic background to express an epileptic phenotype.

## Conclusions

While HCN4 channelopathies have been mostly associated so far with a variety of different types of inheritable cardiac arrhythmias, the data presented here show that dysfunctional HCN4 channels can also be involved in human epileptogenesis, demonstrating for the first time that a loss-of-function HCN4 mutation can lead to increased neuronal excitability.

While failure to detect in our patients other mutations in almost 100 genes previously associated with epilepsy pathogenesis and/or HCN channel function strengthens the hypothesis of the involvement of the R550C HCN4 mutation in the disease, it is important to stress that our data are not sufficient to prove a causative role. We can however hypothesize that this mutation acts as a predisposing factor, in the background of other still undetermined contributing epigenetic and/or genetic conditions.

Since HCN4 expression is high during infancy and declines with age, our data support the intriguing possibility that this HCN isoform has a more specific role in infantile forms of epilepsy. Knowledge of genetic predisposition to myoclonic epilepsy of infancy would be useful in the clinical setting to facilitate early diagnosis and provide a simple and safe tool for prompt therapeutic approach. In order to broaden the analysis of potential pathogenic mechanisms and novel pharmacological targets, it would be important to include *HCN4* in the screening of the genetic factors contributing to infantile epilepsies.

## Author Contributions

JD, ABarbuti and DD conceived and planned the experiments. GC, RM and MBonzanni performed functional experiments and analysis. BC and CG carried out genetic analysis of patients. JD, CF, SF, LC, FR, EF, AL, AG, CC and TG: recruitment and clinical characterization of patients. TG-R analyzed cardiac characterization of patients. GC, JD, ABarbuti and DD wrote the article. ABucchi and MBaruscotti contributed to the interpretation of the results. All authors provided critical feedback and helped shape the research.

## Conflict of Interest Statement

The authors declare that the research was conducted in the absence of any commercial or financial relationships that could be construed as a potential conflict of interest.

## Footnotes

^1^https://www.ncbi.nlm.nih.gov/projects/SNP/

^2^browser.1000genomes.org

^3^evs.gs.washington.edu

^4^http://exac.broadinstitute.org/

^5^http://gnomad.broadinstitute.org/

^6^http://gnomad.broadinstitute.org/

## References

[B1] Arsov T. Mullen S. A. Rogers S. Phillips A. M. Lawrence K. M. Damiano J. A.. (2012). Glucose transporter 1 deficiency in the idiopathic generalized epilepsies. Ann. Neurol. 72, 807–815. 10.1002/ana.23702 23280796

[B2] Avitabile D. Crespi A. Brioschi C. Parente V. Toietta G. Devanna P.. (2011). Human cord blood CD34^+^ progenitor cells acquire functional cardiac properties through a cell fusion process. Am. J. Physiol. Heart Circ. Physiol. 300, H1875–1884. 10.1152/ajpheart.00523.2010 21357510

[B3] Baruscotti M. Bottelli G. Milanesi R. DiFrancesco J. C. DiFrancesco D. (2010). HCN-related channelopathies. Pflugers Arch. 460, 405–415. 10.1007/s00424-010-0810-8 20213494

[B4] Baruscotti M. Bucchi A. Milanesi R. Paina M. Barbuti A. Gnecchi-Ruscone T.. (2017). A gain-of-function mutation in the cardiac pacemaker HCN4 channel increasing cAMP sensitivity is associated with familial Inappropriate Sinus Tachycardia. Eur. Heart J. 38, 280–288. 10.1093/eurheartj/ehv582 28182231

[B5] Baruscotti M. Bucchi A. Viscomi C. Mandelli G. Consalez G. Gnecchi-Rusconi T.. (2011). Deep bradycardia and heart block caused by inducible cardiac-specific knockout of the pacemaker channel gene *Hcn4*. Proc. Natl. Acad. Sci. U S A 108, 1705–1710. 10.1073/pnas.1010122108 21220308 PMC3029742

[B6] Battefeld A. Rocha N. Stadler K. Bräuer A. U. Strauss U. (2012). Distinct perinatal features of the hyperpolarization-activated non-selective cation current *I_h_* in the rat cortical plate. Neural Dev. 7:21. 10.1186/1749-8104-7-21 22694806 PMC3518177

[B7] Becker F. Reid C. A. Hallmann K. Tae H. S. Phillips A. M. Teodorescu G.. (2017). Functional variants in *HCN4* and *CACNA1H* may contribute to genetic generalized epilepsy. Epilepsia Open 2, 334–342. 10.1002/epi4.12068 29588962 PMC5862120

[B8] Benarroch E. E. (2013). HCN channels: function and clinical implications. Neurology 80, 304–310. 10.1212/WNL.0b013e31827dec42 23319474

[B9] Bender R. A. Brewster A. Santoro B. Ludwig A. Hofmann F. Biel M.. (2001). Differential and age-dependent expression of hyperpolarization-activated, cyclic nucleotide-gated cation channel isoforms 1-4 suggests evolving roles in the developing rat hippocampus. Neuroscience 106, 689–698. 10.1016/s0306-4522(01)00314-1 11682156 PMC3084019

[B10] Biel M. Wahl-Schott C. Michalakis S. Zong X. (2009). Hyperpolarization-activated cation channels: from genes to function. Physiol. Rev. 89, 847–885. 10.1152/physrev.00029.2008 19584315

[B100] Bonzanni M. DiFrancesco J. C. Milanesi R. Campostrini G. Castellotti B. Bucchi A. (2018). A novel *de novo* HCN1 loss-of-function mutation in genetic generalized epilepsy causing increased neuronal excitability. Neurobiol. Dis. 118, 55–63. 10.1016/j.nbd.2018.06.012 29936235

[B11] Brewster A. L. Chen Y. Bender R. A. Yeh A. Shigemoto R. Baram T. Z. (2007). Quantitative analysis and subcellular distribution of mRNA and protein expression of the hyperpolarization-activated cyclic nucleotide-gated channels throughout development in rat hippocampus. Cereb. Cortex 17, 702–712. 10.1093/cercor/bhk021 16648453 PMC3100721

[B12] Brown H. F. DiFrancesco D. Noble S. J. (1979). How does adrenaline accelerate the heart? Nature 280, 235–236. 10.1038/280235a0 450140

[B13] Castaldo P. del Giudice E. M. Coppola G. Pascotto A. Annunziato L. Taglialatela M. (2002). Benign familial neonatal convulsions caused by altered gating of KCNQ2/KCNQ3 potassium channels. J. Neurosci. 22:RC199. 10.1523/JNEUROSCI.22-02-j0003.2002 11784811 PMC6758678

[B14] Chung W. K. Shin M. Jaramillo T. C. Leibel R. L. LeDuc C. A. Fischer S. G.. (2009). Absence epilepsy in *apathetic*, a spontaneous mutant mouse lacking the h channel subunit, HCN2. Neurobiol. Dis. 33, 499–508. 10.1016/j.nbd.2008.12.004 19150498 PMC2643333

[B15] Craven K. B. Zagotta W. N. (2004). Salt bridges and gating in the COOH-terminal region of HCN2 and CNGA1 channels. J. Gen. Physiol. 124, 663–677. 10.1085/jgp.200409178 15572346 PMC2234033

[B16] Crino P. B. (2007). Gene expression, genetics, and genomics in epilepsy: some answers, more questions. Epilepsia 48, 42–50. 10.1111/j.1528-1167.2007.01066.x 17571352

[B17] Dibbens L. M. Reid C. A. Hodgson B. Thomas E. A. Phillips A. M. Gazina E.. (2010). Augmented currents of an HCN2 variant in patients with febrile seizure syndromes. Ann. Neurol. 67, 542–546. 10.1002/ana.21909 20437590 PMC3383007

[B18] DiFrancesco D. (1993). Pacemaker mechanisms in cardiac tissue. Annu. Rev. Physiol. 55, 455–472. 10.1146/annurev.physiol.55.1.455 7682045

[B19] DiFrancesco D. (2010a). Funny channel-based pacemaking. Heart Rhythm 7, 276–279. 10.1016/j.hrthm.2009.10.030 20022815

[B20] DiFrancesco D. (2010b). The role of the funny current in pacemaker activity. Circ. Res. 106, 434–446. 10.1161/CIRCRESAHA.109.208041 20167941

[B21] DiFrancesco D. (2013). Funny channel gene mutations associated with arrhythmias. J. Physiol. 591, 4117–4124. 10.1113/jphysiol.2013.253765 23507880 PMC3779106

[B22] DiFrancesco D. (2015). HCN4, sinus bradycardia and atrial fibrillation. Arrhythm. Electrophysiol. Rev. 4, 9–13. 10.15420/aer.2015.4.1.9 26835093 PMC4711571

[B25] DiFrancesco J. C. Barbuti A. Milanesi R. Coco S. Bucchi A. Bottelli G.. (2011). Recessive loss-of-function mutation in the pacemaker HCN2 channel causing increased neuronal excitability in a patient with idiopathic generalized epilepsy. J. Neurosci. 31, 17327–17337. 10.1523/JNEUROSCI.3727-11.2011 22131395 PMC6623833

[B26] DiFrancesco J. C. DiFrancesco D. (2015). Dysfunctional HCN ion channels in neurological diseases. Front. Cell. Neurosci. 6:174. 10.3389/fncel.2015.00071 25805968 PMC4354400

[B23] DiFrancesco D. Noble D. (2012). The funny current has a major pacemaking role in the sinus node. Heart Rhythm 9, 299–301. 10.1016/j.hrthm.2011.09.021 21925134

[B27] DiFrancesco J. C. Novara F. Zuffardi O. Forlino A. Gioia R. Cossu F.. (2015). TREX1 C-terminal frameshift mutations in the systemic variant of retinal vasculopathy with cerebral leukodystrophy. Neurol. Sci. 36, 323–330. 10.1007/s10072-014-1944-9 25213617

[B24] DiFrancesco D. Tortora P. (1991). Direct activation of cardiac pacemaker channels by intracellular cyclic AMP. Nature 351, 145–147. 10.1038/351145a0 1709448

[B28] DiFrancesco J. C. Sestini R. Cossu F. Bolognesi M. Sala E. Mariani S.. (2014). Novel neurofibromatosis type 2 mutation presenting with status epilepticus. Epileptic Disord. 16, 132–137. 10.1684/epd.2014.0647 24667735

[B29] Doan T. N. Kunze D. L. (1999). Contribution of the hyperpolarization-activated current to the resting membrane potential of rat nodose sensory neurons. J. Physiol. 514, 125–138. 10.1111/j.1469-7793.1999.125af.x 9831721 PMC2269051

[B30] Dyhrfjeld-Johnsen J. Morgan R. J. Soltesz I. (2009). Double trouble? Potential for hyperexcitability following both channelopathic up- and downregulation of *I_h_* in epilepsy. Front. Neurosci. 3, 25–33. 10.3389/neuro.01.005.2009 19753094 PMC2695388

[B31] Epi4K Consortium Epilepsy Phenome/Genome Project. (2017). Ultra-rare genetic variation in common epilepsies: a case-control sequencing study. Lancet Neurol. 16, 135–143. 10.1016/S1474-4422(16)30359-3 28102150

[B32] Gambardella A. Marini C. (2009). Clinical spectrum of SCN1A mutations. Epilepsia 50, 20–23. 10.1111/j.1528-1167.2009.02115.x 19469841

[B33] He C. Chen F. Li B. Hu Z. (2014). Neurophysiology of HCN channels: from cellular functions to multiple regulations. Prog. Neurobiol. 112, 1–23. 10.1016/j.pneurobio.2013.10.001 24184323

[B34] Helbig I. Scheffer I. E. Mulley J. C. Berkovic S. F. (2008). Navigating the channels and beyond: unravelling the genetics of the epilepsies. Lancet Neurol. 7, 231–245. 10.1016/S1474-4422(08)70039-5 18275925

[B35] Huang Z. Walker M. C. Shah M. M. (2009). Loss of dendritic HCN1 subunits enhances cortical excitability and epileptogenesis. J. Neurosci. 29, 10979–10988. 10.1523/JNEUROSCI.1531-09.2009 19726656 PMC2744118

[B36] Hughes D. I. Boyle K. A. Kinnon C. M. Bilsland C. Quayle J. A. Callister R. J.. (2013). HCN4 subunit expression in fast-spiking interneurons of the rat spinal cord and hippocampus. Neuroscience 237, 7–18. 10.1016/j.neuroscience.2013.01.028 23357121 PMC3620460

[B37] Kanyshkova T. Pawlowski M. Meuth P. Dubé C. Bender R. A. Brewster A. L.. (2009). Postnatal expression pattern of HCN channel isoforms in thalamic neurons: relationship to maturation of thalamocortical oscillations. J. Neurosci. 29, 8847–8857. 10.1523/JNEUROSCI.0689-09.2009 19587292 PMC2768285

[B38] Li M. Maljevic S. Phillips A. M. Petrovski S. Hildebrand M. S. Burgess R.. (2018). Gain-of-function HCN2 variants in genetic epilepsy. Hum. Mutat. 39, 202–209. 10.1002/humu.23357 29064616

[B39] Ludwig A. Budde T. Stieber J. Moosmang S. Wahl C. Holthoff K.. (2003). Absence epilepsy and sinus dysrhythmia in mice lacking the pacemaker channel HCN2. EMBO J. 22, 216–224. 10.1093/emboj/cdg032 12514127 PMC140107

[B40] Lupica C. R. Bell J. A. Hoffman A. F. Watson P. L. (2001). Contribution of the hyperpolarization-activated current *I_h_* to membrane potential and GABA release in hippocampal interneurons. J. Neurophysiol. 86, 261–268. 10.1152/jn.2001.86.1.261 11431507

[B41] Meuth S. G. Kanyshkova T. Meuth P. Landgraf P. Munsch T. Ludwig A.. (2006). Membrane resting potential of thalamocortical relay neurons is shaped by the interaction among TASK3 and HCN2 channels. J. Neurophysiol. 96, 1517–1529. 10.1152/jn.01212.2005 16760342

[B42] Milanesi R. Baruscotti M. Gnecchi-Ruscone T. DiFrancesco D. (2006). Familial sinus bradycardia associated with a mutation in the cardiac pacemaker channel. N. Engl. J. Med. 354, 151–157. 10.1056/nejmx060027 16407510

[B43] Moosmang S. Biel M. Hofmann F. Ludwig A. (1999). Differential distribution of four hyperpolarization-activated cation channels in mouse brain. Biol. Chem. 380, 975–980. 10.1515/bc.1999.121 10494850

[B44] Nakamura Y. Shi X. Numata T. Mori Y. Inoue R. Lossin C.. (2013). Novel HCN2 mutation contributes to febrile seizures by shifting the channel’s kinetics in a temperature-dependent manner. PLoS One 8:e80376. 10.1371/journal.pone.0080376 24324597 PMC3851455

[B45] Nava C. Dalle C. Rastetter A. Striano P. de Kovel C. G. Nabbout R.. (2014). *De novo* mutations in *HCN1* cause early infantile epileptic encephalopathy. Nat. Genet. 46, 640–645. 10.1038/ng.2952 24747641

[B46] Commission on Classification and Terminology of the International League Against Epilepsy. (1989). Proposal for revised classification of epilepsies and epileptic syndromes. Epilepsia 30, 389–399. 10.1111/j.1528-1157.1989.tb05316.x 2502382

[B47] Nolan M. F. Dudman J. T. Dodson P. D. Santoro B. (2007). HCN1 channels control resting and active integrative properties of stellate cells from layer II of the entorhinal cortex. J. Neurosci. 27, 12440–12451. 10.1523/JNEUROSCI.2358-07.2007 18003822 PMC6673323

[B48] Notomi T. Shigemoto R. (2004). Immunohistochemical localization of *I_h_* channel subunits, HCN1–4, in the rat brain. J. Comp. Neurol. 471, 241–276. 10.1002/cne.11039 14991560

[B49] Pape H. C. (1996). Queer current and pacemaker: the hyperpolarization-activated cation current in neurons. Annu. Rev. Physiol. 58, 299–327. 10.1146/annurev.physiol.58.1.299 8815797

[B50] Qu J. Altomare C. Bucchi A. DiFrancesco D. Robinson R. B. (2002). Functional comparison of HCN isoforms expressed in ventricular and HEK 293 cells. Pflugers Arch. 444, 597–601. 10.1007/s00424-002-0860-7 12194012

[B51] Reid C. A. Phillips A. M. Petrou S. (2012). HCN channelopathies: pathophysiology in genetic epilepsy and therapeutic implications. Br. J. Pharmacol. 165, 49–56. 10.1111/j.1476-5381.2011.01507.x 21615728 PMC3252965

[B52] Robinson R. B. Siegelbaum S. A. (2003). Hyperpolarization-activated cation currents: from molecules to physiological function. Annu. Rev. Physiol. 65, 453–480. 10.1146/annurev.physiol.65.092101.142734 12471170

[B53] Santoro B. Chen S. Luthi A. Pavlidis P. Shumyatsky G. P. Tibbs G. R.. (2000). Molecular and functional heterogeneity of hyperpolarization-activated pacemaker channels in the mouse CNS. J. Neurosci. 20, 5264–5275. 10.1523/JNEUROSCI.20-14-05264.2000 10884310 PMC6772310

[B54] Santoro B. Lee J. Y. Englot D. J. Gildersleeve S. Piskorowski R. A. Siegelbaum S. A.. (2010). Increased seizure severity and seizure-related death in mice lacking HCN1 channels. Epilepsia 51, 1624–1627. 10.1111/j.1528-1167.2010.02554.x 20384728 PMC2952649

[B55] Schulze-Bahr E. Neu A. Friederich P. Kaupp U. B. Breithardt G. Pongs O.. (2003). Pacemaker channel dysfunction in a patient with sinus node disease. J. Clin. Invest. 111, 1537–1545. 10.1172/jci16387 12750403 PMC155041

[B56] Seo H. Seol M. J. Lee K. (2015). Differential expression of hyperpolarization-activated cyclic nucleotide-gated channel subunits during hippocampal development in the mouse. Mol. Brain 8:13. 10.1186/s13041-015-0103-4 25761792 PMC4352274

[B58] Shao Y. Chen Y. (2017). Pathophysiology and clinical utility of non-coding RNAs in epilepsy. Front. Mol. Neurosci. 10:249. 10.3389/fnmol.2017.00249 28848386 PMC5554344

[B57] Shah M. M. Huang Z. Martinello K. (2013). HCN and K_V_7 (M−) channels as targets for epilepsy treatment. Neuropharmacology 69, 75–81. 10.1016/j.neuropharm.2012.03.005 22446478 PMC4104618

[B59] Shi W. Wymore R. Yu H. Wu J. Wymore R. T. Pan Z.. (1999). Distribution and prevalence of hyperpolarization-activated cation channel (HCN) mRNA expression in cardiac tissues. Circ. Res. 85, e1–e6. 10.1161/01.res.85.1.e1 10400919

[B60] Stoenica L. Wilkars W. Battefeld A. Stadler K. Bender R. Strauss U. (2013). HCN1 subunits contribute to the kinetics of *I_h_* in neonatal cortical plate neurons. Dev. Neurobiol. 73, 785–797. 10.1002/dneu.22104 23821600

[B61] Striano P. Weber Y. G. Toliat M. R. Schubert J. Leu C. Chaimana R.. (2012). GLUT1 mutations are a rare cause of familial idiopathic generalized epilepsy. Neurology 78, 557–562. 10.1212/WNL.0b013e318247ff54 22282645

[B62] Surges R. Brewster A. L. Bender R. A. Beck H. Feuerstein T. J. Baram T. Z. (2006). Regulated expression of HCN channels and cAMP levels shape the properties of the h current in developing rat hippocampus. Eur. J. Neurosci. 24, 94–104. 10.1111/j.1460-9568.2006.04880.x 16882011 PMC2919221

[B63] Tang B. Sander T. Craven K. B. Hempelmann A. Escayg A. (2008). Mutation analysis of the hyperpolarization-activated cyclic nucleotide-gated channels HCN1 and HCN2 in idiopathic generalized epilepsy. Neurobiol. Dis. 29, 59–70. 10.1016/j.nbd.2007.08.006 17931874 PMC2709210

[B64] Thomas R. H. Berkovic S. F. (2014). The hidden genetics of epilepsy-a clinically important new paradigm. Nat. Rev. Neurol. 10, 283–292. 10.1038/nrneurol.2014.62 24733163

[B65] Verkerk A. O. Wilders R. (2015). Pacemaker activity of the human sinoatrial node: an update on the effects of mutations in HCN4 on the hyperpolarization-activated current. Int. J. Mol. Sci. 16, 3071–3094. 10.3390/ijms16023071 25642760 PMC4346881

[B66] Wahl-Schott C. Fenske S. Biel M. (2014). HCN channels: new roles in sinoatrial node function. Curr. Opin. Pharmacol. 15, 83–90. 10.1016/j.coph.2013.12.005 24441197

[B67] Xu X. Vysotskaya Z. V. Liu Q. Zhou L. (2010). Structural basis for the cAMP-dependent gating in the human HCN4 channel. J. Biol. Chem. 285, 37082–37091. 10.1074/jbc.M110.152033 20829353 PMC2978636

